# Dirac fermion heating, current scaling, and direct insulator-quantum Hall transition in multilayer epitaxial graphene

**DOI:** 10.1186/1556-276X-8-360

**Published:** 2013-08-22

**Authors:** Fan-Hung Liu, Chang-Shun Hsu, Chiashain Chuang, Tak-Pong Woo, Lung-I Huang, Shun-Tsung Lo, Yasuhiro Fukuyama, Yanfei Yang, Randolph E Elmquist, Chi-Te Liang

**Affiliations:** 1 Graduate Institute of Applied Physics, National Taiwan University, Taipei 106, Taiwan; 2 Department of Physics, National Taiwan University, Taipei 106, Taiwan; 3 National Institute of Advanced Industrial Science and Technology (AIST), Tsukuba, Ibaraki 305-8568, Japan; 4 National Institute of Standards and Technology (NIST), Gaithersburg, MD 20899, USA

**Keywords:** Graphene, Magnetoresistivity measurements, Direct insulator-quantum Hall transition

## Abstract

We have performed magnetotransport measurements on multilayer epitaxial graphene. By increasing the driving current *I* through our graphene devices while keeping the bath temperature fixed, we are able to study Dirac fermion heating and current scaling in such devices. Using zero-field resistivity as a self thermometer, we are able to determine the effective Dirac fermion temperature (*T*_DF_) at various driving currents. At zero field, it is found that *T*_DF_ ∝ *I*^≈1/2^. Such results are consistent with electron heating in conventional two-dimensional systems in the plateau-plateau transition regime. With increasing magnetic field *B*, we observe an *I*-independent point in the measured longitudinal resistivity *ρ*_xx_ which is equivalent to the direct insulator-quantum Hall (I-QH) transition characterized by a temperature-independent point in *ρ*_xx_. Together with recent experimental evidence for direct I-QH transition, our new data suggest that such a transition is a universal effect in graphene, albeit further studies are required to obtain a thorough understanding of such an effect.

## Background

Graphene, which is an ideal two-dimensional (2D) system, has been attracting worldwide interest since its discovery in 2004 [1]. While the sizes of mechanically exfoliated graphene are limited, its ultrahigh quality allows one to observe fascinating physical phenomena such as ambipolar characteristics [1], anomalous integer quantum Hall steps [1], Berry's phase [2,3], and fractional quantum Hall effect [4-6]. On the other hand, graphene prepared by chemical vapor deposition (CVD) and epitaxial graphene can be used for potential device applications because the sizes of these systems should allow realization of wafer-scale integrated circuits based on graphene [7].

When a charge system is appreciably heated by a driving current, the equilibrium between the phonons and the charges collapses. In this situation, effective charge temperature (*T*_*c*_) can be substantially higher than lattice temperature (*T*_*L*_) [8]. This interesting physical phenomenon is normally called the charge heating effect. In some cases, there exists a simple effective charge temperature-current relation *T*_*c*_ ∝ *I*^*α*^, where *α* is an exponent that depends on charge-phonon scattering [8]. It is now well established that the two-bath model can be used to describe charge heating and charge energy loss rate by charge-phonon scattering [8]. The charge heating effect has become increasingly important as device dimensions are reduced and charge mobility is increased [9]. In particular, Dirac fermion heating in graphene is an important physical phenomenon since it affects thermal dissipation and heat management in modern electronics [10] and low-temperature applications such as quantum resistance metrology [11].

Insulator-quantum Hall (I-QH) transition [12-15] is an interesting physical phenomenon in the field of 2D physics. Especially, a direct transition from an insulator to a high Landau level filling factor *ν* ≥ 3 QH state which is normally described as the direct I-QH transition continues to attract interest [16-18]. Very recently, experimental evidence for direct I-QH transition in epitaxial monolayer graphene [19] and in mechanically exfoliated multilayer graphene [20] has been reported. In order to further study direct I-QH transition in the graphene-based system, one may wish to investigate Dirac fermion heating in graphene. Moreover, it is a fundamental issue to see if a *current*-independent point in the longitudinal resistivity when the bath temperature is fixed exists since such a point should be equivalent to the direct I-QH transition. Furthermore, one could probe current scaling on both sides of the direct I-QH transition to further study Dirac fermion-phonon scattering as well as Dirac fermion-Dirac fermion scattering, both of which are very fundamental physical phenomena.

In this paper, we report magnetotransport measurements on multilayer epitaxial graphene of few layers obtained under conditions which favor controlled growth at high temperatures [21]. Dirac fermion heating in the high current limit is studied. It is found that in the low magnetic field regime, the effective Dirac fermion temperature obeys a simple power law *T*_DF_ ∝ *I*^≈0.5^. Such results suggest that the Dirac fermion-phonon scattering rate 1/*τ*_DFP_ ~ *T*^2^, consistent with those in conventional 2D electron systems. With increasing magnetic field, interestingly, a current-independent point in the longitudinal resistivity is observed. It was demonstrated that such a point corresponds to the direct I-QH transition characterized by a *T*-independent point in *ρ*_xx_. This result is further supported by the vastly different *I* dependences for both sides of the I-QH transition. Our new experimental results, together with recent experimental results [19,20], indicate that direct I-QH transition is a universal effect in graphene. We suggest that further experimental and theoretical studies are required to obtain a complete picture for direct I-QH transition in graphene-based devices.

## Methods

A controlled sublimation method was used for graphene growth on a 6H-SiC (0001) surface [16]. First, the SiC substrate was cleaned using a standard procedure for substrate cleaning [21]. Second, the optically polished Si-face surface was placed face-to-face with a polished graphite disk (FTG) and arranged such that uniform Newton rings were observed in fluorescent light [21]. The optically finished substrate surfaces resulted in a higher rate of SiC decomposition compared to chemical–mechanical processed (CMP) surfaces and created multiple graphene layers.

The epitaxial growth process was controlled by annealing in a sequence of temperature ramp and dwell stages in Ar background gas at a pressure slightly higher than 1 atm using a commercial furnace. The substrates were first dehydrated and cleaned in the furnace at 725°C for approximately 16 h. The temperature was ramped to 1,200°C for 30 min and then ramped at 100°C/min for graphene growth at a temperature (dwell time) of 1,850°C (45 min; samples 1 and 2) or 1,950°C (30 min; samples 3 and 4). The temperatures were measured and controlled using molybdenum-sheathed type ‘C’ thermocouples.

**Figure 1:**
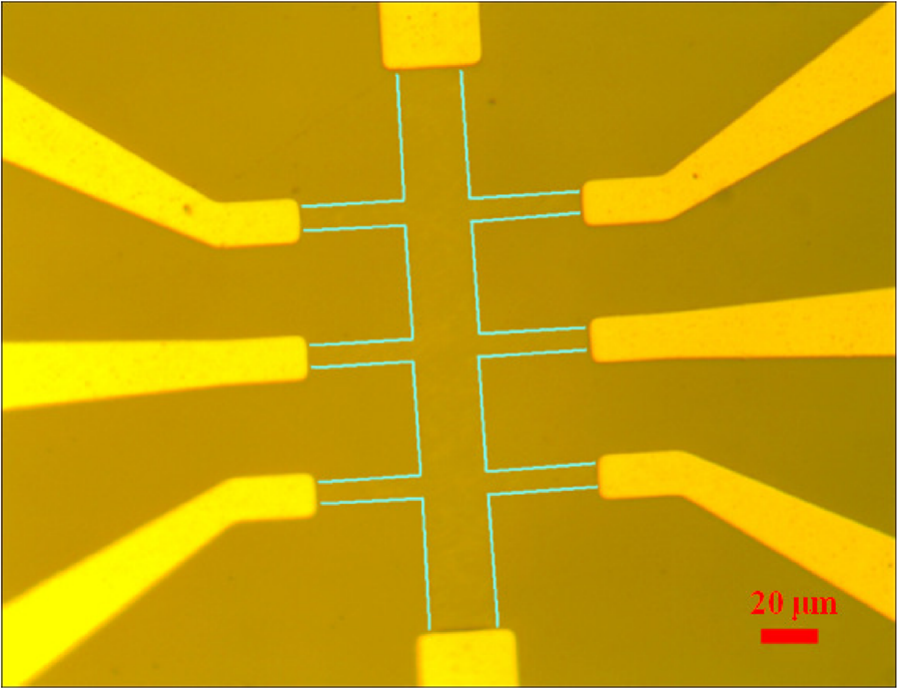
**Optical microscopy image of Hall bar shows *L *= 100 and *W *= 25 μm.** The green lines indicate the edges of the Hall bar.

When the samples were taken out of the furnace, they were imaged by tapping-mode atomic force microscopy (AFM). They were then shipped from NIST to National Taiwan University, where they were patterning into Hall bars by standard photolithography using reactive ion etch in O^2^ plasma (see Figure 1 with size ratio *L*/*W* = 4). The pleats on the surface show that multilayer graphene was grown over most of the 6H-SiC (0001) surface [22]. Optically polished substrates produce much thicker graphene for the same processing conditions compared to that grown on CMP surfaces. The roughness of the optically polished surface provides much more off-axis surface area, relative to the (0001) atomic plane, and this accounts for the faster growth rate. The TEM images are taken from samples grown under the same conditions. Comparing the AFM images with TEM imaging performed on other samples, we would estimate that the 1,850°C samples have four to five layers of graphene and the 1,950°C samples have five to six layers. All four-terminal electrical measurements were carried out using dc constant-current sources and multimeters.

## Results and discussion

**Figure 2:**
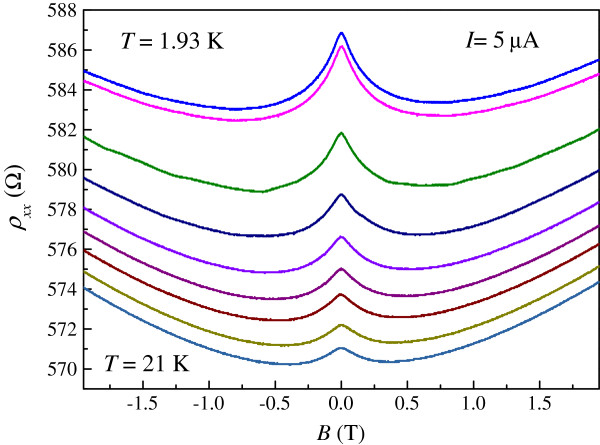
**The magnetoresistivity measurements *ρ***_**xx **_***(B) *at different temperatures *T.*** From top to bottom: *T* = 1.93, 1.98, 4, 6, 8, 10, 12, 15, 18, and 21 K.

Figure 2 shows the magnetoresistivity measurements *ρ*_xx_ (*B*) at various temperatures. Negative magnetoresistivity centered at *B* = 0 can be ascribed to suppression of weak localization by a magnetic field applied perpendicular to the graphene plane. The weak localization effect in graphene is interesting as, initially, it was suggested that weak localization is strongly suppressed in exfoliated graphene flakes while normally pronounced positive magnetoresistivity centered at *B* = 0 is observed [23]. Later it was shown that the weak localization effect depends strongly on the chirality of the graphene system [24]. In epitaxial graphene, pronounced negative magnetoresistivity is often observed, allowing studies of weak localization in graphene-based systems [25]. As shown in Figure 2, the observed negative magnetoresistivity becomes less pronounced with increasing temperature.

**Figure 3:**
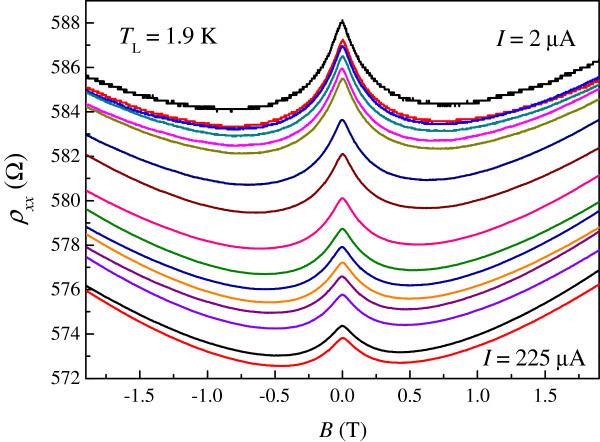
**Magnetoresistivity measurements *ρ***_**xx **_***(B) *at driving currents *I.*** The lattice temperature is constantly fixed at *T* ≈ 1.9 K. From top to bottom: *I* = 2, 3, 5, 7, 8.5, 10, 20, 30, 50, 70, 85, 100, 125, 150, 200, and 225 μA, respectively.

**Figure 4:**
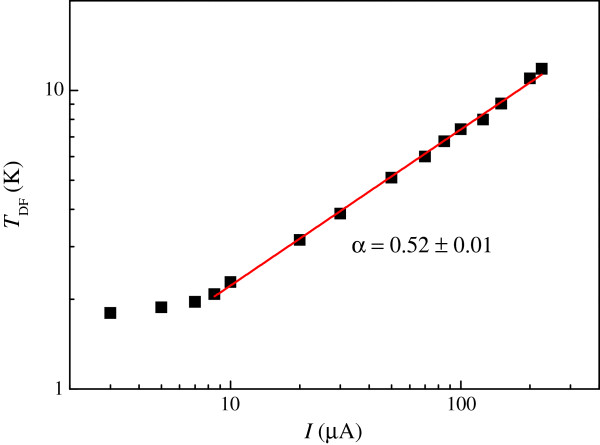
**Effective Dirac fermion temperature *T***_**DF **_**versus driving current *I *on a log-log scale.** The red line corresponds to the best fit in the high-current regime. The exponent in the *T*_DF_-*I* relation is given as *α* = 0.52 ± 0.01. The error stems from interpolation of the magnetoresistivity data.

Figure 3 shows the magnetoresistivity measurements *ρ*_xx_ (*B*) at various driving currents with the lattice temperature at ≈2 K. The negative magnetoresistivity observed centered at zero field shows a strong dependence on current and is suppressed at higher currents. We suggest that increasing the measurement temperature in the low current limit is equivalent to increasing the current while keeping the lattice temperature constant at approximately ≈2 K. These results can be ascribed to Dirac fermion heating in which the equilibrium between the phonons and Dirac fermion collapses. Using the zero-field resistivity of our device as a self thermometer, we are able to determine the effective Dirac fermion temperature at various driving currents. Such results are shown in Figure 4. In the low current limit, *T*_DF_ is approximately *I*-independent, suggesting that the lattice temperature is equal to *T*_DF_. In the high current limit, *T*_DF_ ∝ *I*^≈0.52^. The measured exponent in the *T*_DF_-*I* relation is close to one half. Such a result is consistent with heating effects observed in various 2D systems in the plateau-plateau transition regime [26,27]. Here we follow the seminal work of Scherer and co-workers [26]. The inelastic scattering length can be given by

(1) $$l_{\mathrm{in}}\propto T^{\frac{-p}{2}}$$

where *p* is the exponent related to inelastic scattering. The effective electron temperature is given by the energy acquired by the electron diffusing along the distance *l*_in_ in the electric field *E*. Therefore,

(2) $$k_{B}T_{e}\approx \mathrm{eE}l_{\mathrm{in}}$$

Upon inserting Equation 2 and *E* ~ *J* ~ *I*, we have

(3) $$T_{e}\approx I^{\frac{2}{2+p}}$$

If *p* = 2 [10,25], then the exponent in the temperature-scaling relation is 0.5 [21,26-28] which is consistent with our experimental results obtained on Dirac fermions. We note that our experimental results are equivalent to a *T*^4^ dependence of energy loss rate for Dirac fermions as calculated [29] and observed in epitaxial, CVD-grown and exfoliated graphene [10,30]. It is worth pointing out that previous results are obtained in the plateau-plateau transition regime [26,27,31] and Shubnikov-de Haas region [10], which is in contrast with our case in the weak insulating regime where Landau quantization is not significant. Nevertheless, our data indeed indicate such a universal exponent at approximately 0.5 for heating in various 2D systems. Moreover, our results suggest that the Dirac fermion-phonon scattering rate 1/*τ*_DFP_ is proportional to *T*^2^. It is worth noting that enhanced mobility can be achieved in semiconductor quantum wires [32] and in semiconducting graphene nanoribbons [33] by a high dc electric field. Such interesting results are highly desirable for practical applications in narrow graphene devices in the high current limit.

**Figure 5:**
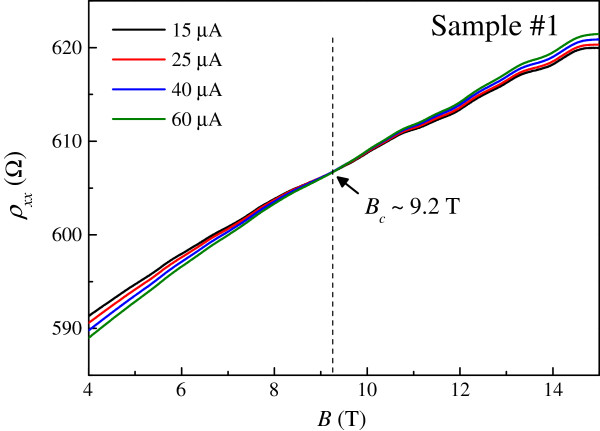
**Magnetoresistivity measurements *ρ***_**xx **_***(B) *at various driving currents *I.*** The lattice temperature is constantly fixed at *T* ≈ 2 K.

**Figure 6:**
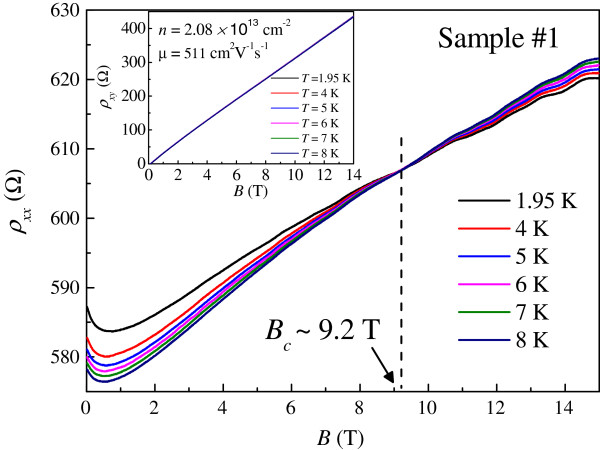
**The magnetoresistivity measurements *ρ***_**xx **_***(B) *at different *T *for sample 1.** The inset shows the Hall measurements *ρ*_xy_ (*B*) at different *T* for sample 1.

**Figure 7:**
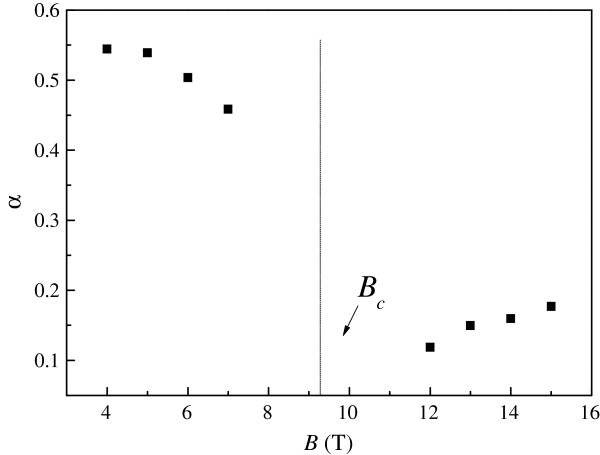
**The determined exponent *α *in the power law *T***_**DF**_ ∝ ***I***^***α***^**versus magnetic field *B.***

In order to further study the observed Dirac fermion heating effects, we have extended our measurements to higher magnetic fields. Such results are shown in Figure 5. Interestingly, a current-independent point in *ρ*_xx_ is observed. The observed fixed point is reminiscent of the I-QH transition in graphene [19,20]. In order to confirm this interpretation, as shown in Figure 6, we perform magnetoresistivity measurements *ρ*_xx_ (*B*) at various temperatures in the low current limit to ensure thermal equilibrium between phonons and Dirac fermions. The same crossing point in *ρ*_xx_ at *B*_c_ ≈ 9.2 T is indeed observed. For *B* <*B*_c_, the resistivity decreases with increasing temperature, as is characteristic of an insulator [17]. For *B* >*B*_c_, the resistivity increases with increasing temperature, showing a QH conductor behavior [17]. In the high magnetic field regime, some weak oscillatory features can be ascribed to Shubnikov-de Haas oscillations in disordered graphene. However, their amplitudes are weak; therefore, it is not possible to extract important physical quantities such as the quantum mobility and effective mass in our system. The Landau level filling factor at the crossing point is estimated to be ≈94. Therefore, we have observed compelling evidence for the direct I-QH in disordered epitaxial graphene. Using the measured *ρ*_xx_ as a thermometer for Dirac fermions, we are able to determine *T*_DF_ and the exponent in the *T*_DF_-*I* relation at different magnetic fields as shown in Figure 7. Close to *B*_c_, the temperature dependence of *ρ*_xx_ is so weak that reliable determination of *T*_DF_ cannot be obtained. We note that in the insulating regime *B* <*B*_c_, the exponent is again close to one half, consistent with the results at *B* = 0. In the QH-like regime, the exponent is about 0.15 which is significantly smaller than one half. Such vastly different exponents observed in the two regimes provide further experimental evidence for the direct I-QH transition in disordered epitaxial graphene. We note that defining physically an effective temperature for non-thermal-equilibrium electrons is non-trivial [34], which is not always a scaling relation based on a linear response theory for perturbative thermal-equilibrium states. Therefore, further studies are required for a better understanding of our results.

In studying multilayer epitaxial graphene, top gating is difficult since depositing a dielectric layer is difficult and the top layers would screen the electric fields. Back gating is impractical because it would require SiC substrate thinning. Therefore, in order to further study the observed direct I-QH transition, we choose to study various samples with different classical mobilities (see Additional file 1). In all cases, an approximately *T*-independent point in *ρ*_xx_ is observed. The approximated *T*-independent Hall results suggest that Dirac fermion-Dirac fermion interactions are not significant in all our devices [35-38]. The crossing point and some other physical quantities are listed in Table 1. We note that for the same numbers of layer, the crossing field *B*_c_ is lower when the mobility *μ* is higher, consistent with the results obtained in conventional GaAs-based 2D systems [39,40]. Moreover, the spin degree of freedom does not play an important role in the observed direct I-QH transition [41-45]. The dependence of the crossing magnetic field on the number of layers and sample does not seem to show a trend and thus requires further studies.

**Table 1:** Sample parameters

	**Sample 1**	**Sample 2**	**Sample 3**	**Sample 4**
*ρ* (Ω)	583	520	443	367
*n* (10^13^ cm^−2^)	2.08	1.98	2.16	2.44
*μ* (cm^2^/V.S)	511	605	651	694
*B*_c_ (T)	9.2	4.2	6.0	5.7
*v*_c_	94	194	148	178
*ρ*_xx_/*ρ*_xy_ at *B*_c_	2.1	3.7	2.5	2.8
*μB*_c_	0.47	0.25	0.39	0.40

Samples 1 and 2 were from the same chip, processed at 1,850°C for 45 min; the former is close to the edge, and the later is near the center. Samples 3 and 4 were also from the same chip, processed at 1,950°C for 30 min; the former is close to the center, and the latter is near the edge. Lower resistivity near the edge is expected in the FTG process; near the center the graphene growth is suppressed because of the higher concentration of Si vapor.

At the crossing fields, the corresponding Landau filling factors are much larger than 2. Therefore, we have observed direct I-QH transition in all our devices [17-20]. It was argued that for direct I-QH transition in conventional semiconductor-based 2D systems, near the crossing field, *ρ*_xx_ is approximately *ρ*_xy_, and the product of *μB*_c_ is close to 1 [46]. However, in all our devices, *ρ*_xx_/*ρ*_xy_ is much greater than 1, and *μB*_c_ is always smaller than 1. Therefore, our data suggest that further studies are required to obtain a thorough understanding of the direct I-QH transition not only in conventional 2D systems but also in disordered graphene. The observation of a current-independent point in *ρ*_xx_ which corresponds to its temperature-independent counterpart suggests that applying a high current is equivalent to heating up the graphene lattice.

## Conclusions

In conclusion, we have presented magnetoresistivity measurements on multilayer epitaxial graphene. It is found that a relation between the effective Dirac fermion temperature and the driving current can be given by *T*_DF_ ∝ *I*^≈0.5^ in the low magnetic field regime. With increasing magnetic field, an *I*-independent point in *ρ*_xx_ is observed which is equivalent to its *T*-independent counterpart in the low current limit. Evidence for direct I-QH transition has been reported in four different graphene samples. Near the crossing field where the longitudinal resistivity is approximately *T*-independent, *ρ*_xx_ is at least two times larger than *ρ*_xy_. Moreover, the product of Drude mobility and *B*_c_ is smaller than 1. We suggest that further studies are required to obtain a complete understanding of direct I-QH transition in disordered graphene.

## Competing interests

The authors declare that they have no competing interests.

## Authors’ contributions

FHL, CSH, CC, TPW, and LIH performed the experiments. FHL, YF, YY, and REE fabricated the device. REE and CTL coordinated the project. TPW and STL provided key interpretation of the data. FHL and CTL drafted the paper. All the authors read and approved the final manuscript.

## Supplementary Material

Additional file 1: Figure S1 The magnetoresistivity measurements *ρ*_xx_ (*B*) at different *T* for sample 2. The inset shows the Hall measurements *ρ*_xy_ (*B*) at different *T* for sample 2. **Figure S2** The magnetoresistivity measurements *ρ*_xx_ (*B*) at different *T* for sample 3. The inset shows the Hall measurements *ρ*_xy_ (*B*) at different *T* for sample 3. **Figure S3** The magnetoresistivity measurements *ρ*_xx_ (*B*) at different *T* for sample 4. The inset shows the Hall measurements *ρ*_xy_ (*B*) at different *T* for sample 4. Click here for file
