# Supplementary material for: Dirac fermion heating, current scaling, and direct insulator-quantum Hall transition in multilayer epitaxial graphene
Source: Nanoscale Res Lett. 2013 Aug 22;8(1):360. doi: 10.1186/1556-276X-8-360 (PMC3765374; doi:10.1186/1556-276X-8-360)
Supplement: Additional file 1: Figure S1 — The magnetoresistivity measurements ρxx (B) at different T for sample 2. The inset shows the Hall measurements ρxy (B) at different T for sample 2. Figure S2 The magnetoresistivity measurements ρxx (B) at different T for sample 3. The inset shows the Hall measurements ρxy (B) at different T for sample 3. Figure S3 The magnetoresistivity measurements ρxx (B) at different T for sample 4. The inset shows the Hall measurements ρxy (B) at different T for sample 4. [file 1556-276X-8-360-S1.docx]

**Additional file**

Additional file 1: Figure S1 The magnetoresistivity measurements *ρ*_xx_ (*B*) at different *T* for Sample 2. The inset shows the Hall measurements *ρ*_xy_ (*B*) at different *T* for Sample 2.

Additional file 1: Figure S2 The magnetoresistivity measurements *ρ*_xx_ (*B*) at different *T* for Sample 3. The inset shows the Hall measurements *ρ*_xy_ (*B*) at different *T* for Sample 3.

Additional file 1: Figure S3 The magnetoresistivity measurements *ρ*_xx_ (*B*) at different *T* for Sample 4. The inset shows the Hall measurements *ρ*_xy_ (*B*) at different *T* for Sample 4.
